# Diagnostic value of integrating salivary and blood miRNAs for pancreatic cancer detection

**DOI:** 10.3389/fonc.2025.1642727

**Published:** 2025-10-20

**Authors:** Parker Wilson, Taichiro Nonaka

**Affiliations:** ^1^ School of Medicine, Louisiana State University Health Shreveport, Shreveport, LA, United States; ^2^ Department of Cellular Biology and Anatomy, Louisiana State University Health Sciences Center, Shreveport, LA, United States; ^3^ Feist-Weiller Cancer Center, Louisiana State University Health Shreveport, Shreveport, LA, United States

**Keywords:** pancreatic cancer, microRNAs, circulating biomarker, liquid biopsy, saliva diagnostics

## Abstract

**Background:**

Pancreatic cancer remains one of the most lethal malignancies due to its late-stage diagnosis and limited treatment options. Conventional diagnostic methods, such as imaging and tissue biopsy, often lack sensitivity in early-stage detection and are invasive, limiting their widespread application. There is an urgent need for non-invasive, highly accurate biomarkers to facilitate early diagnosis and improve patient outcomes. Circulating microRNAs (miRNAs) have emerged as promising liquid biopsy biomarkers, offering the potential for early detection through minimally invasive methods. This meta-analysis aims to evaluate the diagnostic performance of blood- and saliva-derived miRNAs in detecting pancreatic cancer.

**Methods:**

A systematic search of PubMed, Web of Science, and Scopus databases identified 350 relevant studies. After removing duplicates and applying eligibility criteria, 27 studies with 1,496 patients were included. These studies contained 168 sub-studies, each assessing the diagnostic potential of individual miRNAs. Quality assessment was conducted using the QUADAS-2 tool, and meta-analysis was performed using a random-effects model. Sensitivity, specificity, diagnostic odds ratio (DOR), and summary receiver operating characteristic (SROC) curves were analyzed to determine diagnostic performance.

**Results:**

Blood-derived miRNAs demonstrated a pooled sensitivity of 0.83 (95% CI: 0.78-0.88) and specificity of 0.87 (95% CI: 0.82-0.91), while saliva-derived miRNAs exhibited slightly higher sensitivity at 0.87 (95% CI: 0.84-0.90) and specificity at 0.86 (95% CI: 0.82-0.89). The combined analysis yielded a sensitivity of 0.86 (95% CI: 0.84-0.89) and specificity of 0.85 (95% CI: 0.83-0.88). The area under the curve (AUC) for blood-derived miRNAs was 0.92 (95% CI: 0.89-0.94), whereas saliva-derived miRNAs achieved an AUC of 0.93 (95% CI: 0.90-0.95). The combined analysis resulted in an AUC of 0.92 (95% CI: 0.90-0.94). Diagnostic odds ratios were 33.40 (95% CI: 17.88-62.37) for blood-derived miRNAs, 39.94 (95% CI: 28.66-55.67) for saliva-derived miRNAs, and 37.04 (95% CI: 27.66-49.60) for the combined dataset.

**Conclusion:**

Both blood- and saliva-derived miRNAs exhibit strong diagnostic performance for pancreatic cancer, with saliva-derived miRNAs demonstrating slightly higher accuracy. These findings support the potential of circulating miRNAs as non-invasive biomarkers that could address the current limitations in pancreatic cancer diagnosis. Further large-scale, well-controlled studies are warranted to confirm these results and optimize their clinical application.

## 1 Introduction

Pancreatic cancer remains one of the most lethal malignancies worldwide, characterized by a high incidence and mortality rate (1). According to recent statistics, pancreatic cancer ranks as the third leading cause of cancer-related deaths in the United States, with a five-year survival rate of around 13% (2). The poor prognosis is primarily due to the aggressive nature of the disease, late-stage diagnosis, and limited therapeutic options (3). The disease is often asymptomatic in its early stages, leading to delayed diagnosis and a subsequent lack of effective curative treatment.

The incidence of pancreatic cancer has been increasing over the past few decades (4). While its exact cause remains elusive, several well-recognized risk factors have been identified, including smoking, obesity, chronic pancreatitis, diabetes mellitus, and genetic predisposition (4). Smoking is one of the most significant modifiable risk factors, with long-term smokers exhibiting a substantially higher risk (5–7). Chronic inflammation in conditions such as pancreatitis promotes cellular changes that increase the likelihood of malignant transformation (8, 9). Additionally, genetic syndromes such as familial atypical multiple mole melanoma (FAMMM) syndrome and BRCA mutations further contribute to an individual’s susceptibility to pancreatic cancer (10–12).

Pathologically, pancreatic ductal adenocarcinoma (PDAC) is the most prevalent histological type, accounting for more than 90% of cases (13). PDAC is characterized by an abundant desmoplastic stroma, which contributes to its resistance to treatment (13). Genetically, pancreatic cancer is driven by key mutations in oncogenes and tumor suppressor genes (14). The most commonly mutated gene in PDAC is KRAS, which is found in over 90% of cases and plays a crucial role in tumorigenesis by promoting uncontrolled cell proliferation (14). Other frequently mutated genes include TP53, CDKN2A, and SMAD4, all of which influence tumor progression, cell cycle regulation, and metastatic potential (14).

The diagnosis of pancreatic cancer relies on a combination of imaging techniques, histopathological examination, and serological biomarkers (15). Endoscopic ultrasound-guided fine-needle aspiration (EUS-FNA) is the gold standard for obtaining tissue for histological confirmation (16, 17). Additionally, imaging modalities such as computed tomography (CT), magnetic resonance imaging (MRI), and positron emission tomography (PET) scans provide valuable information to assess tumor stage and resectability (18–20). Serum biomarkers such as carbohydrate antigen 19-9 (CA19-9) are frequently used to assist in diagnosis and prognosis (21). However, CA19–9 lacks sufficient sensitivity and specificity, particularly in early-stage pancreatic cancer, leading to false-negative and false-positive results (22). Moreover, CA19–9 is also elevated in benign conditions such as pancreatitis and obstructive jaundice, further limiting its diagnostic utility (23–25). Given these limitations, there is an urgent need for more reliable, non-invasive biomarkers that can detect pancreatic cancer at an early stage, improve diagnostic accuracy, and aid in disease monitoring.

Liquid biopsy has recently emerged as a promising diagnostic approach in oncology, providing a minimally invasive and repeatable method for detecting cancer-related molecular changes (26). Unlike traditional tissue biopsies, which are invasive and often impractical for repeated sampling, liquid biopsies allow for continuous monitoring of tumor dynamics, enabling a more personalized approach to treatment and disease surveillance (27). Liquid biopsy enables the analysis of circulating biomarkers, including circulating tumor DNA (ctDNA), circulating tumor cells (CTCs), and circulating microRNAs (miRNAs) from bodily fluids such as blood and saliva (27, 28). Each of these biomarkers plays a unique role in cancer detection. ctDNA originates from apoptotic and necrotic tumor cells and contains genetic mutations representative of the tumor’s mutational landscape, making it a valuable tool for detecting genetic alterations, tracking tumor evolution, and monitoring treatment response (29). CTCs, on the other hand, are intact tumor cells that have detached from the primary tumor and entered the circulation, with their presence strongly associated with metastatic potential (30). However, their rarity poses a challenge for clinical implementation.

Among circulating biomarkers, miRNAs have gained increasing attention due to their stability, regulatory functions, and potential as cancer biomarkers (31). miRNAs are small, non-coding RNAs that modulate gene expression by targeting messenger RNAs (mRNAs) for degradation or translational repression. Dysregulated miRNA expression is a hallmark of cancer, contributing to oncogenesis by regulating pathways involved in proliferation, apoptosis, and metastasis (32). miRNAs are released into bodily fluids through different mechanisms (33). Some circulate as free-floating miRNAs, which are unbound molecules in the bloodstream and susceptible to rapid degradation (34). Others exist as protein-bound miRNAs, associated with RNA-binding proteins such as Argonaute (AGO) complexes, which protect them from degradation (35, 36). A particularly promising category is exosomal miRNAs, which are encapsulated within extracellular vesicles such as exosomes, protecting them from enzymatic degradation and facilitating intercellular communication (37–39). Among these, exosomal miRNAs are particularly valuable as biomarkers due to their stability and tumor-specific expression patterns (40, 41).

In cancer, miRNAs function either as oncogenes, known as oncomiRs, or as tumor suppressors, depending on their target genes. miRNA profiling studies have demonstrated unique miRNA expression signatures in pancreatic cancer, distinguishing malignant from benign conditions (42). Certain miRNAs, such as miR-21, miR-155, and miR-196a, are upregulated in pancreatic cancer and have been implicated in tumor progression, while others, such as miR-200c and miR-375, are downregulated and play roles in tumor suppression (43, 44).

A particularly novel approach to liquid biopsy involves the analysis of saliva-derived exosomal miRNAs (45). Exosomal miRNAs originating from pancreatic tumors can reach the saliva through circulation, offering a non-invasive and convenient diagnostic method (45, 46). Saliva-based diagnostics have gained prominence, especially during the COVID-19 pandemic, when saliva testing became widely adopted for viral detection (47). Saliva collection is non-invasive, painless, and enables high patient compliance, making it an attractive option for cancer screening and disease monitoring (47).

Saliva-based liquid biopsy offers several advantages over blood-based biomarker detection (48). Since saliva collection does not require needle punctures or invasive procedures, it reduces patient discomfort and anxiety, leading to higher compliance. Additionally, saliva samples can be collected multiple times, enabling longitudinal disease monitoring without the need for repeated venipuncture. The ease of at-home collection also facilitates early disease detection and broadens accessibility to diagnostic testing.

Given the potential of miRNAs as cancer biomarkers, our study aims to evaluate the diagnostic value of circulating miRNAs in the blood and saliva of pancreatic cancer patients through meta-analysis. By systematically analyzing existing data, we seek to determine the reliability and clinical applicability of miRNA-based liquid biopsy for early detection and disease monitoring of pancreatic cancer. This research has the potential to contribute to the development of more accessible, accurate, and patient-friendly diagnostic tools, ultimately improving outcomes for pancreatic cancer patients.

## 2 Materials and methods

### 2.1 Search strategy

**Figure 1:**
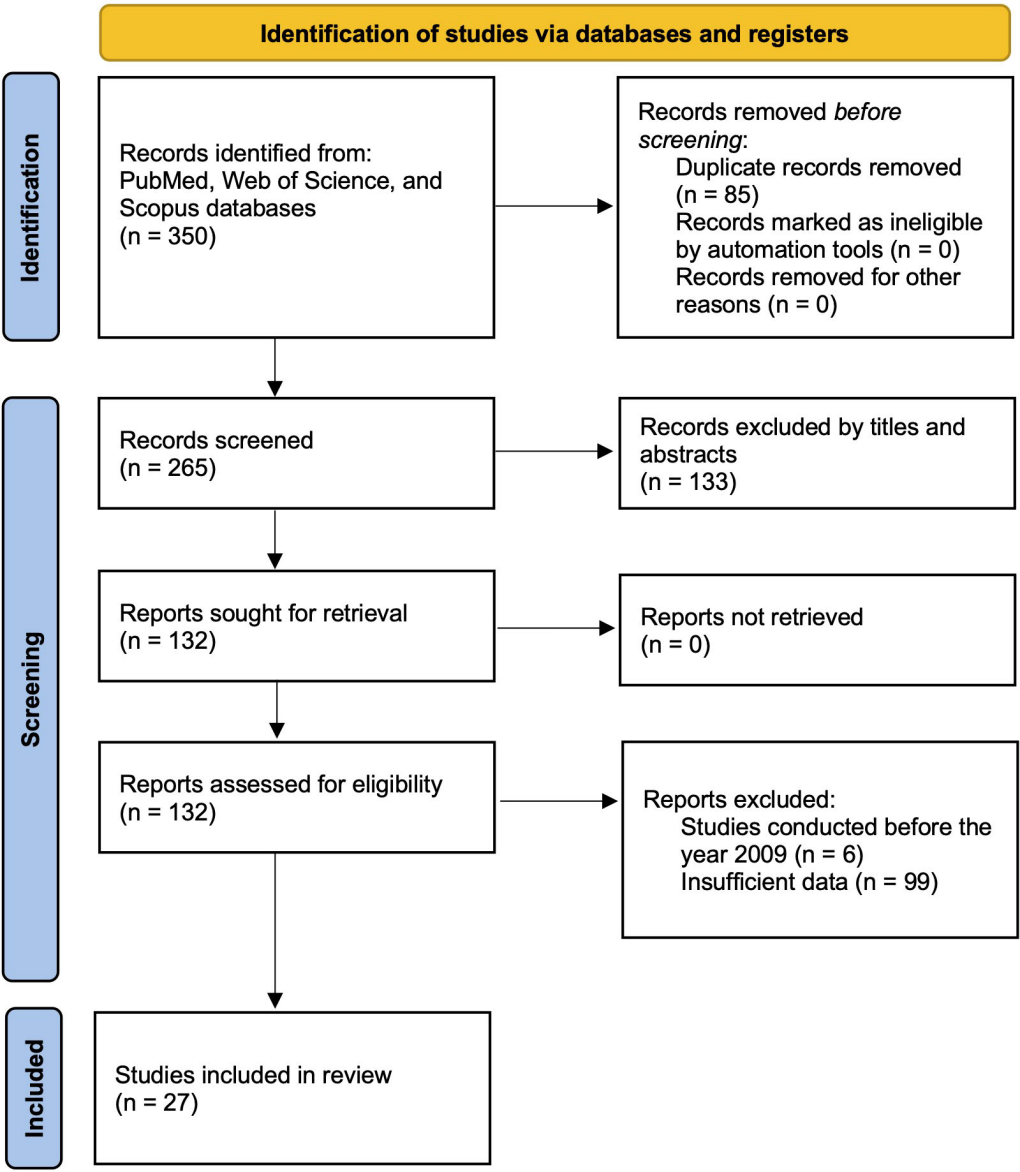
PRISMA flow diagram illustrating the study selection process.

A systematic and comprehensive literature search was conducted across multiple major databases, including PubMed, Web of Science, and Scopus, to identify relevant studies investigating miRNA liquid biomarkers in various biological fluids, such as blood, urine, saliva, and other bodily fluids. The search strategy incorporated both Medical Subject Headings (MeSH) terms and free-text keywords related to “pancreatic cancer”, “microRNA”, “liquid biopsy”, “diagnosis”, and specific fluid types (i.e., plasma, serum, or saliva) to ensure comprehensive retrieval of relevant articles. MeSH is standardized indexing terms used in databases such as PubMed to improve the precision of search results. The search was restricted to studies published between 2009 and 2024. Studies were included if they provided or allowed calculation of essential diagnostic performance metrics, including sensitivity, specificity, area under the curve (AUC), and changes in miRNA expression levels. The study selection process, including the number of articles screened at each stage, is illustrated in the PRISMA flow diagram (**Figure 1**) (49).

### 2.2 Eligibility criteria

Studies were selected based on predefined inclusion and exclusion criteria. Only studies involving human subjects were considered eligible. Inclusion criteria required that studies be peer-reviewed, non-duplicated, published within the last 15 years, and contain sufficient data for diagnostic accuracy assessment. Studies were included if they provided explicit data on miRNA expression changes and diagnostic performance measures, either reported directly or calculable from the given results for constructing a 2x2 contingency table, including true positives (TP), true negatives (TN), false positives (FP), and false negatives (FN). The inclusion criterion requiring a 2x2 contingency table ensures that each study provides sufficient data to calculate sensitivity, specificity, and likelihood ratios, which are essential for meta-analytic pooling of diagnostic accuracy metrics. Additionally, eligible studies needed to include both case and control groups for comparative analysis. Exclusion criteria comprised studies involving animal models, cell lines, review articles, case reports, conference abstracts, and studies lacking sufficient quantitative data.

### 2.3 Data extraction and quality assessment

For each eligible study, two independent reviewers extracted data using a standardized data extraction form. The extracted data included study characteristics such as author names, year of publication, study design, sample size for both cases and controls, and the type of biological fluid analyzed. Additionally, miRNA biomarker profiles were collected, including specific miRNAs investigated, fold change in expression, and detection methods such as qRT-PCR, microarray, or RNA sequencing. Although studies using microarray or RNA sequencing were initially screened, all studies ultimately included in the meta-analysis employed qRT-PCR for miRNA quantification. Diagnostic accuracy metrics, including TP, TN, FP, FN, sensitivity, specificity, and AUC values with their respective 95% confidence intervals (CI), were also recorded. Quality assessment of the included studies was conducted using the Quality Assessment of Diagnostic Accuracy Studies (QUADAS-2) tool, implemented via RevMan (v5.4) (50). The risk of bias and applicability concerns were evaluated across 4 domains: patient selection, index test, reference standard, and flow and timing.

### 2.4 Statistical analysis

All statistical analyses were performed using STATA (v18.0) software. A random-effects model was applied to calculate pooled estimates for sensitivity, specificity, diagnostic likelihood ratios (DLR positive and negative), diagnostic score (DS), and diagnostic odds ratio (DOR) with corresponding 95% confidence intervals. The use of a bivariate random-effects meta-analysis model ensures that smaller or more variable studies are weighted appropriately and helps prevent overestimation of pooled diagnostic performance. Summary receiver operating characteristic (SROC) curves were generated, and the area under the curve (AUC) was calculated to assess the overall diagnostic accuracy of circulating miRNAs. The statistical significance of results was determined using p-values, with a threshold of p < 0.05 considered statistically significant. Heterogeneity among studies was assessed using Cochrane’s Q test and I^2^ statistics, where an I^2^ value greater than 50% was considered indicative of substantial heterogeneity (51). To investigate potential publication bias, Deeks’ funnel plot asymmetry test was performed, with a p-value of less than 0.05 indicating the presence of publication bias. These methodological approaches ensured the inclusion of high-quality studies, minimized bias, and provided a rigorous framework for evaluating the diagnostic performance of circulating miRNA biomarkers.

### 2.5 Visualization of diagnostic and biological significance of miRNAs using Sankey plots

To comprehensively visualize the diagnostic characteristics and biological relevance of individual miRNAs, we constructed interactive Sankey plots as an extension of our meta-analysis (**Figure 8**). The primary aim was to illustrate how frequently reported circulating miRNAs in pancreatic cancer relate to diagnostic accuracy (sensitivity and specificity) and to highlight their functional involvement in cancer-related signaling pathways. Based on the extracted contingency table data (TP, FP, FN, TN) from each sub-study (n = 168), we calculated miRNA-level sensitivity and specificity. For each unique miRNA, all sub-studies in which it appeared were aggregated, and true positive (TP), false positive (FP), false negative (FN), and true negative (TN) counts were summed. Subsequently, sensitivity and specificity values were categorized into “High (≥ 0.90)”, “Moderate (0.70-0.89)”, or “Low (< 0.70)” groups based on the predefined thresholds. The Sankey plots were generated using Python 3.11.4 with the Plotly 5.20.0 library. Data processing and aggregation were conducted using pandas 2.2.2 and python-docx 1.1.0 to extract structured information from the **Supplementary Table 1**. **Figure 8A** focuses on the most frequently reported miRNAs across included studies (≥2 sub-studies) and those known to be functionally associated with pancreatic cancer signaling pathways (e.g., KRAS, TP53, CDKN2A). This visualization connects each miRNA to its categorized specificity and sensitivity levels, emphasizing those with both empirical and biological support. In contrast, **Figure 8B** highlights a curated subset of miRNAs with established links to major pancreatic cancer-related pathways, such as KRAS-MAPK, TP53-mediated apoptosis, CDKN2A/p16 tumor suppression, and PI3K/AKT survival signaling. Each miRNA is linked to one or more pathways based on previously published mechanistic studies, and subsequently connected to its observed diagnostic specificity in our meta-analysis. These layered visualizations complement the quantitative synthesis presented in the main meta-analysis by offering a systems-level view of miRNA relevance, combining frequency, diagnostic metrics, and biological roles.

## 3 Results

### 3.1 Study selection

A systematic search across PubMed, Web of Science, and Scopus databases yielded 350 studies relevant to circulating miRNAs as liquid biopsy biomarkers for pancreatic cancer diagnosis. After removing duplicates, 265 unique articles remained. A preliminary screening of titles and abstracts led to the exclusion of 133 studies that did not meet the eligibility criteria. The remaining 132 studie s underwent full-text review, which resulted in the exclusion of 6 studies published before 2009. Additionally, 99 studies were removed due to insufficient data for diagnostic performance assessment. Consequently, 27 studies with 1,496 patients were included in the final meta-analysis. Many of the included studies contained multiple sub-studies, each investigating different miRNAs. To ensure methodological rigor, these sub-studies were treated as independent data points, resulting in a total of 168 sub-studies in the final analysis. To distinguish multiple sub-studies from the same paper, numerical designations were appended to the publication year [e.g., Que et al., 2013 (1) and Que et al., 2013 (2)]. These IDs correspond to **Supplementary Table 1**, Serial Numbers 162 and 163. The study selection process is depicted in the PRISMA flow diagram (**Figure 1**), while study characteristics are summarized in **Table 1**.

**Table 1:** Characteristics of the studies included in the meta-analysis.

Author, Year	Location	miRNA	No. of patients	Sample	Ref
Ishige et al., 2020	Japan	miR-1246	41	Serum, Saliva	(60)
Liu et al., 2020	China	miR-196a	40	Plasma	(61)
Wei et al., 2020	China	miR-1246	120	Serum	(62)
Goto et al., 2018	Japan	miR-21	24	Serum	(63)
Kawamura et al., 2018	Japan	miR-21	26	Blood	(64)
Lai et al., 2017	USA	miR-10b, 20a, 21, 30c, 106b, 181a, 483	29	Plasma	(65)
Xu et al., 2017	USA	miR-1246	15	Plasma	(66)
Akamatsu et al., 2016	Japan	miR-7, 34a, 181d, 193b	69	Serum	(67)
Alemar et al., 2016	Brazil	miR-21, 34a	24	Serum	(68)
Deng et al., 2016	China	miR-25	303	Serum	(69)
Hussein et al., 2016	Egypt	miR-22, 642b, 885	35	Plasma	(70)
Machida et al., 2016	Japan	miR-1246, 4644	12	Saliva	(71)
Qu et al., 2016	China	miR-21	56	Serum	(72)
Humeau et al., 2015	France	miR-20a, 21, 23a, 23b, 25, 29c, 92a, 127-5p, 203, 205, 222, 223	7	Saliva	(73)
Komatsu et al., 2015	Japan	miR-223	94	Plasma	(74)
Miyamae et al., 2015	Japan	miR-744	94	Plasma	(75)
Xie et al., 2015	China	miRNA panels*	8	Saliva	(76)
Chen et al., 2014	China	miR-182	109	Plasma	(77)
Cote et al., 2014	USA	miR-10b, 30c, 106b, 155, 212	40	Plasma	(78)
Ganepola et al., 2014	USA	miR-22, 642, 885	11	Plasma	(79)
Gao et al., 2014	China	miR-16	70	Plasma	(80)
Slater et al., 2014	Germany	miR-196a	19	Serum	(81)
Zhang et al., 2014	China	miR-192, 194	70	Serum	(82)
Kawaguchi et al., 2013	Japan	miR-221	47	Plasma	(83)
Que et al., 2013	China	miR-21, 17	22	Serum	(84)
Zhao et al., 2013	China	miR-192	70	Serum	(85)
Wang et al., 2009	USA	miR-21, 155, 196a, 210	28	Plasma	(86)

*miRNA panels consist of 103 miRNAs, the details of which are provided in **Supplementary Table 1**.

### 3.2 Study characteristics

Among the 168 sub-studies, 49 focused on blood-derived miRNAs, whereas 119 investigated saliva-derived miRNAs. The studies exhibited substantial variability in publication year, geographic location, sample size, and the specific miRNAs analyzed. Sample sizes ranged widely from as few as 7 participants to over 300. The dataset was deemed statistically robust due to the large overall sample size, the diverse representation of different populations, and the use of consistent diagnostic criteria across studies, which minimized biases and enhanced the reliability of the pooled results. All sub-studies assessed the diagnostic utility of individual miRNAs rather than panels of multiple miRNAs. This consistency ensured comparability across studies. A detailed summary of sub-studies characteristics, including publication year, sample size, and miRNAs analyzed, is provided in **Supplementary Table 1**.

To enhance transparency and contextual interpretation of diagnostic performance across studies, we extracted and summarized available patient demographic and clinical information from each included study (**Supplementary Table 2**). This included geographic location, cohort setting, miRNA type, mean patient age, sex distribution, cancer type, and disease stage, providing a comprehensive overview of cohort characteristics and potential sources of heterogeneity in the included literature. Patient characteristics across the included studies were heterogeneous in terms of geographic origin, age, sex distribution, and cancer stage. Where reported, the majority of cohorts were hospital-based and involved patients with pancreatic ductal adenocarcinoma (PDAC) across a range of clinical stages (0-IV). Mean patient ages generally ranged from the mid-50s to late 60s, with male predominance in most studies. A detailed breakdown of these attributes outlines key cohort-level characteristics relevant to the interpretation of diagnostic outcomes.

### 3.3 Quality assessment

**Figure 2:**
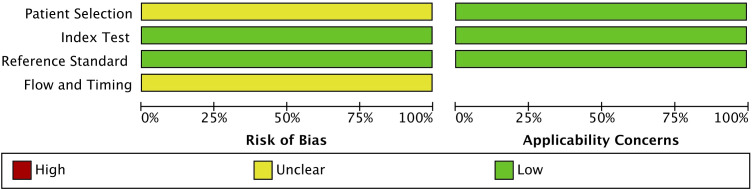
Quality assessment of the included studies using the Quality Assessment of Diagnostic Accuracy Studies 2 (QUADAS-2) tool.

The quality of the included studies was assessed using the QUADAS-2 tool. This assessment covered four key domains: patient selection, index test, reference standard, and flow and timing. The risk of bias and applicability concerns were categorized as low, unclear, or high, with the results summarized in **Figure 2**. The risk of bias assessment indicated that patient selection had an unclear risk of bias (yellow), suggesting possible variability in recruitment methods across studies. However, the index test and reference standard domains were rated as having a low risk of bias (green), ensuring the reliability of the diagnostic tests and gold-standard comparisons. The flow and timing domain also exhibited an unclear risk of bias, reflecting potential inconsistencies in study design or patient follow-up protocols. Regarding applicability concerns, all three domains (i.e., patient selection, index test, and reference standard) were rated as having a low risk, confirming that the included studies were relevant and applicable to the research question. Overall, the QUADAS-2 assessment validated the acceptability of the included studies, supporting the robustness and relevance of the findings.

### 3.4 Meta-analysis

**Figure 3:**
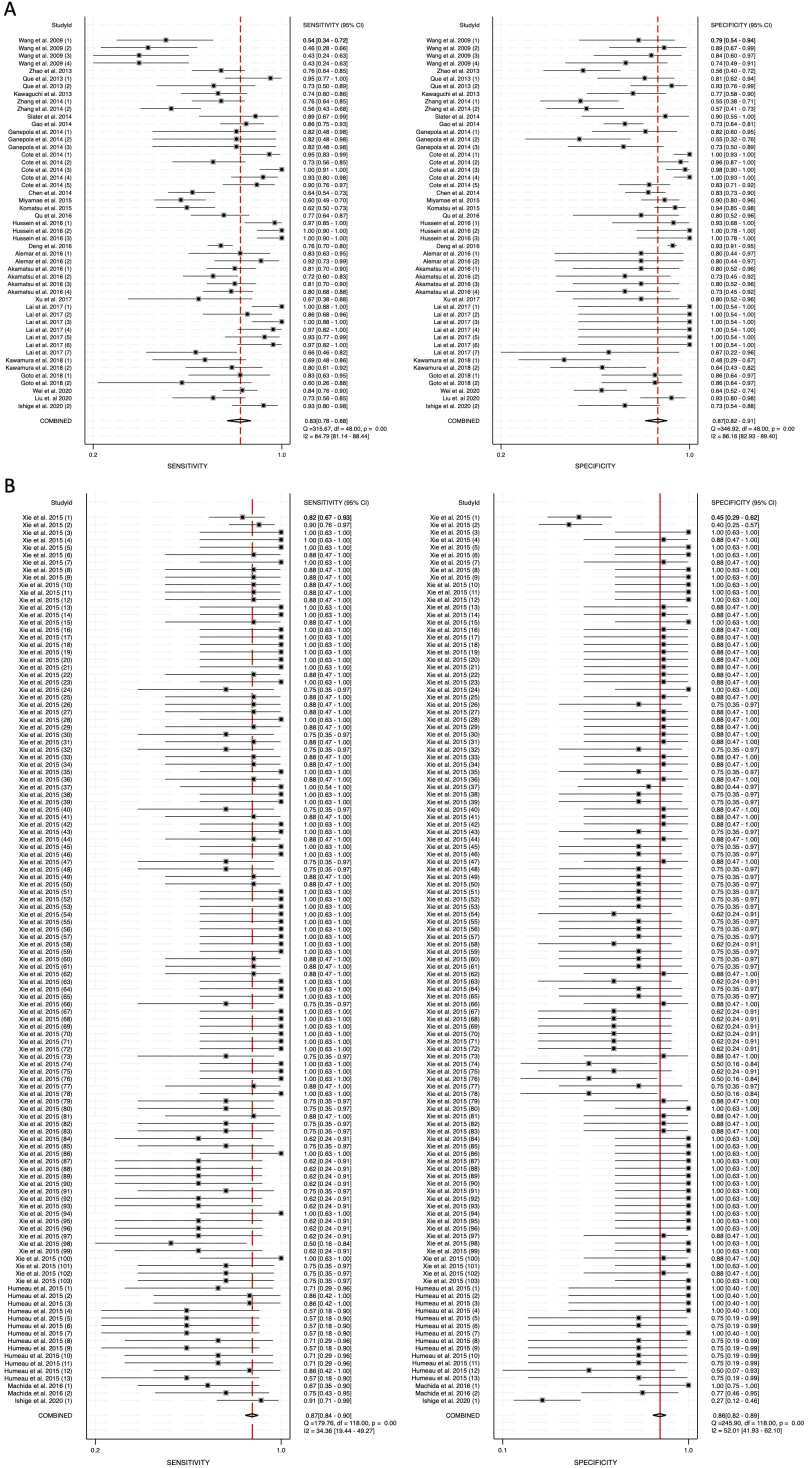
Pooled sensitivity and specificity for circulating miRNA-based diagnostics of pancreatic cancer. **(A)** Blood-derived miRNAs (Sensitivity: 0.83, Specificity: 0.87). **(B)** Saliva-derived miRNAs (Sensitivity: 0.87, Specificity: 0.86).

To assess heterogeneity, the I^2^ statistic was employed. Blood-derived miRNAs demonstrated high heterogeneity, with an I^2^ value of 84.79 for sensitivity and 86.16 for specificity (**Figure 3A**). In contrast, saliva-derived miRNAs exhibited lower heterogeneity, with I^2^ values of 34.36 for sensitivity and 52.01 for specificity (**Figure 3B**). The combined analysis of blood- and saliva-derived miRNAs showed moderate heterogeneity, with I^2^ values of 67.61 for sensitivity and 73.05 for specificity (**Supplementary Figure 1**). Due to this variability, a random-effects model was applied to ensure appropriate pooled estimates.

**Figure 4:**
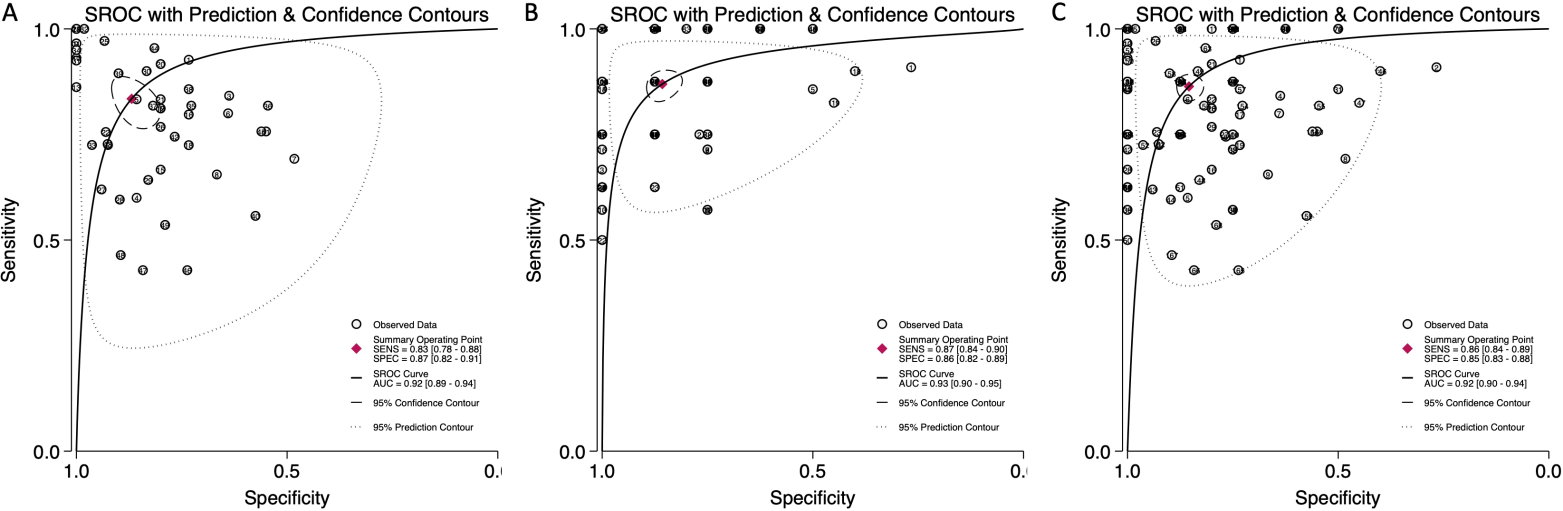
Summary receiver operating characteristic (SROC) curve analysis for circulating miRNA-based pancreatic cancer diagnostics. **(A)** Blood-derived miRNAs (AUC: 0.92, 95% CI: 0.89–0.94). **(B)** Saliva-derived miRNAs alone (AUC: 0.93, 95% CI: 0.90–0.95). **(C)** Combined blood- and saliva-derived miRNAs (AUC: 0.92, 95% CI: 0.90–0.94).

The pooled sensitivity for blood-derived miRNAs was 0.83 (95% CI: 0.78-0.88), while saliva-derived miRNAs demonstrated a slightly higher pooled sensitivity of 0.87 (95% CI: 0.84-0.90) (**Figures 3A, B**). The combined analysis yielded a pooled sensitivity of 0.86 (95% CI: 0.84-0.89) (**Supplementary Figure 1**). Similarly, the pooled specificity was high across all categories, with blood-derived miRNAs achieving 0.87 (95% CI: 0.82-0.91), saliva-derived miRNAs at 0.86 (95% CI: 0.82-0.89), and the combined analysis at 0.85 (95% CI: 0.83-0.88) (**Figure 3**, **Supplementary Figure 1**). To further evaluate diagnostic accuracy, SROC curve analysis was conducted. It provides a composite visualization of the trade-off between sensitivity and specificity across different thresholds and studies, and the area under the curve (AUC) offers a summary metric of overall diagnostic performance. The AUC for blood-derived miRNAs was 0.92 (95% CI: 0.89-0.94), while saliva-derived miRNAs achieved an AUC of 0.93 (95% CI: 0.90-0.95) (**Figures 4A, B**). The combined analysis yielded an AUC of 0.92 (95% CI: 0.90-0.94), demonstrating strong diagnostic performance across all categories (**Figure 4C**).

**Figure 5:**
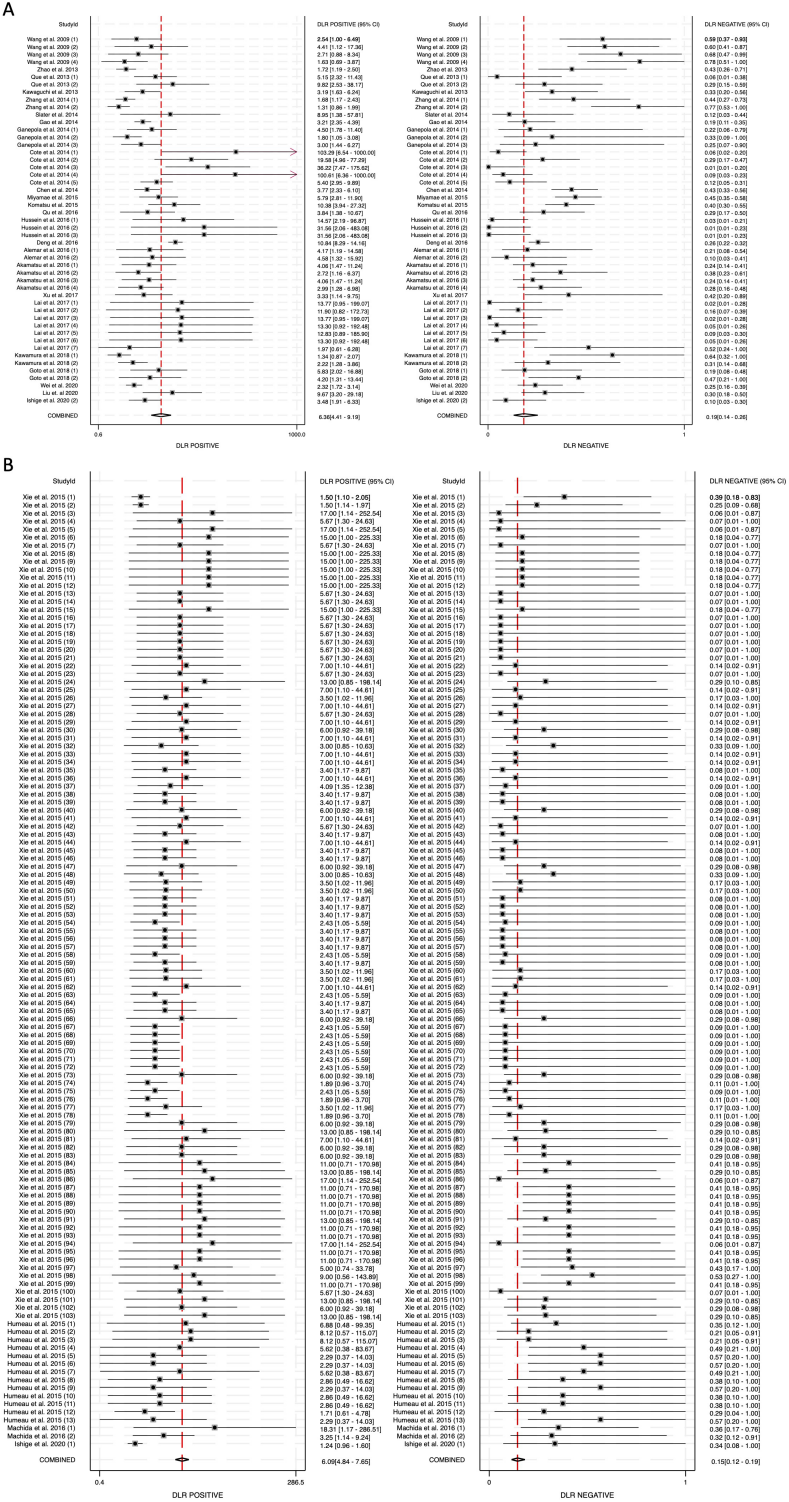
Diagnostic likelihood ratios (DLRs) for circulating miRNA-based pancreatic cancer diagnostics. **(A)** Blood-derived miRNAs (DLR positive: 6.36, DLR negative: 0.19). **(B)** Saliva-derived miRNAs (DLR positive: 6.09, DLR negative: 0.15).

To further assess the clinical utility of circulating miRNAs, we calculated diagnostic likelihood ratios (DLRs), which integrate both sensitivity and specificity to provide a more practical metric for evaluating diagnostic value in real-world settings. The diagnostic likelihood ratio positive (DLR positive) reflects how much the odds of pancreatic cancer increase following a positive test result, while the diagnostic likelihood ratio negative (DLR negative) indicates how much the odds decrease following a negative result. A DLR positive greater than 5 and a DLR negative less than 0.2 are generally considered to provide moderate-to-strong evidence for ruling in or ruling out disease, respectively (52). In our meta-analysis, the DLR positive for blood-derived miRNAs was 6.36 (95% CI: 4.41-9.19), while for saliva-derived miRNAs it was 6.09 (95% CI: 4.84-7.65) (**Figures 5A, B**). The combined analysis showed a DLR positive of 5.92 (95% CI: 4.96-7.05) (**Supplementary Figure 2**). The DLR negative was 0.19 (95% CI: 0.14-0.26) for blood-derived miRNAs, 0.15 (95% CI: 0.12-0.19) for saliva-derived miRNAs, and 0.16 (95% CI: 0.13-0.19) for the combined analysis (**Figure 5**, **Supplementary Figure 2**).

**Figure 6:**
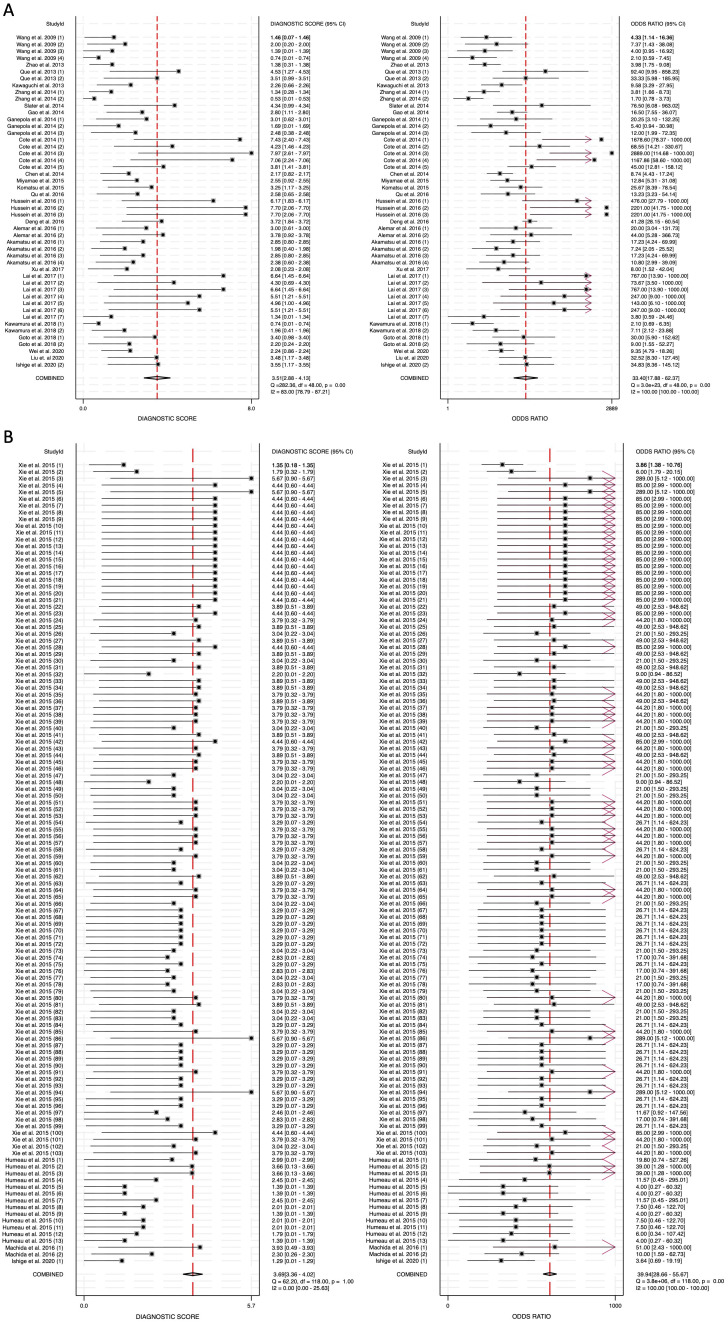
Diagnostic score (DS) and diagnostic odds ratio (DOR) for circulating miRNA-based pancreatic cancer diagnostics. **(A)** Blood-derived miRNAs (DS: 3.51, DOR: 33.40). **(B)** Saliva-derived miRNAs alone (DS: 3.69, DOR: 39.94).

The diagnostic score (DS) was calculated as 3.51 (95% CI: 2.88-4.13) for blood-derived miRNAs, 3.69 (95% CI: 3.36-4.02) for saliva-derived miRNAs, and 3.61 (95% CI: 3.32-3.90) for the combined analysis (**Figure 6**, **Supplementary Figure 3**). Correspondingly, the diagnostic odds ratio (DOR) was 33.40 (95% CI: 17.88-62.37) for blood-derived miRNAs, 39.94 (95% CI: 28.66-55.67) for saliva-derived miRNAs, and 37.04 (95% CI: 27.66-49.60) for the combined dataset (**Figure 6**, **Supplementary Figure 3**). These findings indicate that while saliva-derived miRNAs exhibited slightly superior diagnostic accuracy compared to blood-derived miRNAs, both types demonstrated strong potential as diagnostic tools for pancreatic cancer.

### 3.5 Publication bias

**Figure 7:**
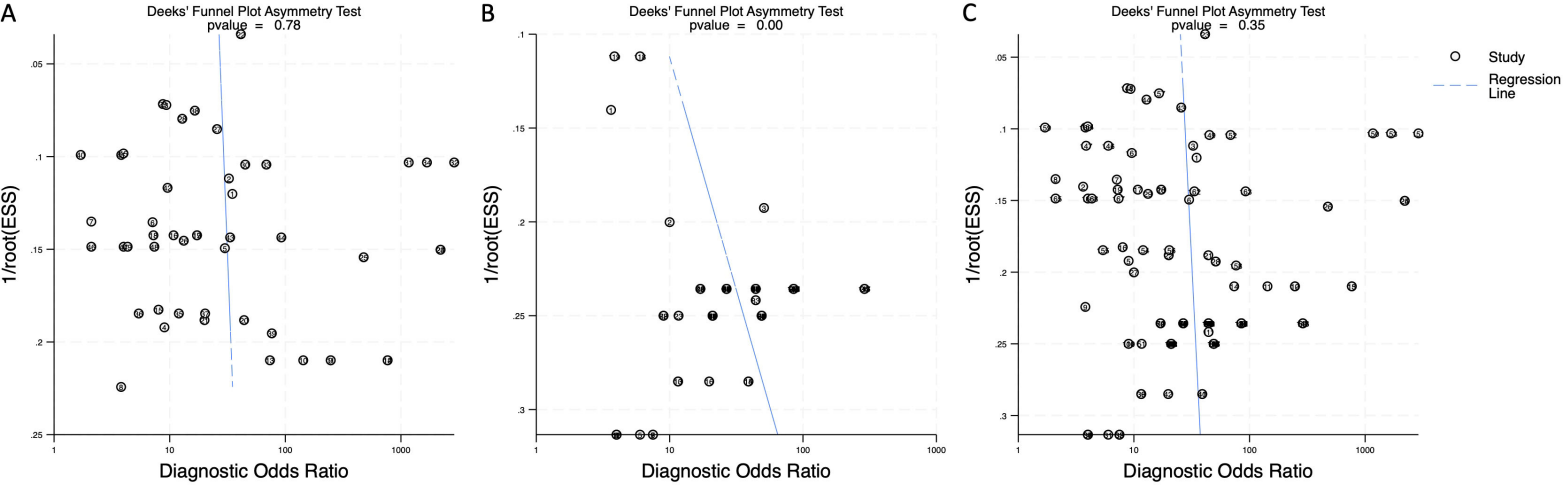
Deeks’ funnel plot assessing publication bias in circulating miRNA-based pancreatic cancer diagnostics. **(A)** Blood-derived miRNAs (p = 0.78). **(B)** Saliva-derived miRNAs alone (p < 0.01). **(C)** Combined blood- and saliva-derived miRNAs (p = 0.35).

To assess potential publication bias, Deeks’ funnel plot asymmetry test was performed. The p-value for blood-derived miRNAs was 0.78, indicating no significant publication bias (**Figure 7A**). Saliva-derived miRNAs exhibited a p-value of less than 0.01, suggesting the presence of publication bias (**Figure 7B**). The combined blood- and saliva-derived miRNA analysis yielded a p-value of 0.35, indicating no significant publication bias (**Figure 7C**). The presence of bias in saliva-derived studies may be attributed to the selective publication of positive results or the underreporting of negative findings. Nonetheless, the absence of significant bias in blood-derived and combined analyses reinforces the reliability of the overall findings.

**Figure 8:**
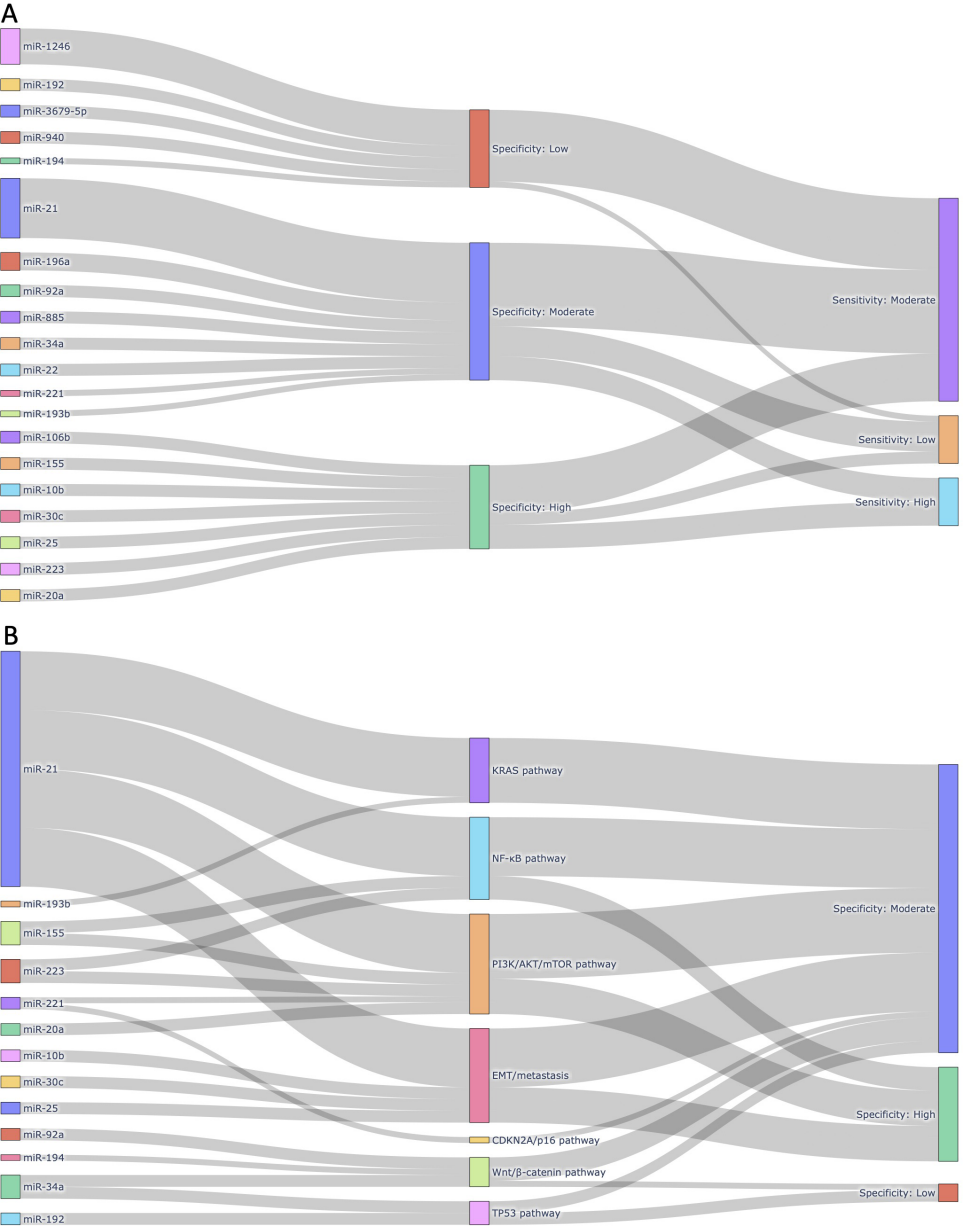
Integrated visualizations of diagnostic performance and biological relevance of circulating miRNAs in pancreatic cancer. **(A)** Sankey plot illustrating the 20 most frequently reported miRNAs across the 168 sub-studies included in this meta-analysis. Each miRNA is linked to its corresponding pooled diagnostic performance category, based on sensitivity and specificity thresholds derived from our meta-analysis. Only miRNAs reported in at least two independent sub-studies and functionally associated with pancreatic cancer were included, highlighting miRNAs with both empirical recurrence and diagnostic potential. **(B)** Sankey plot mapping selected circulating miRNAs to major pancreatic cancer-related molecular pathways in which they are mechanistically implicated. Each miRNA is grouped by its associated pathway (e.g., KRAS, NF-κB, PI3K/AKT/mTOR, EMT/metastasis, CDKN2A/p16, Wnt/β-catenin, TP53), followed by its observed specificity category based on meta-analytic data. This figure emphasizes the biological plausibility of diagnostic miRNAs by linking them to oncogenic or tumor-suppressive signaling cascades relevant to pancreatic tumorigenesis.

### 3.6 Diagnostic performance and biological significance of circulating miRNAs

To further explore the diagnostic utility and biological significance of circulating miRNAs identified through our meta-analysis, we constructed two layered Sankey plots that integrate both quantitative metrics and mechanistic associations (**Figure 8**). **Figure 8A** presents a diagnostic landscape of the 20 most frequently reported miRNAs, each mapped to its corresponding specificity and sensitivity category (**Figure 8A**). These miRNAs were selected based on two criteria: 1) appearance in at least two independent sub-studies, and 2) known involvement in pancreatic cancer-related molecular pathways. This dual criterion ensured inclusion of both empirically robust and biologically plausible markers. The most frequently reported miRNA, miR-21, was associated with moderate sensitivity and specificity, aligning with its well-documented role in oncogenic processes including KRAS and PI3K/AKT signaling. Other highly ranked miRNAs, such as miR-196a, miR-92a, and miR-1246, also exhibited moderate-to-high diagnostic performance.

**Figure 8B** focuses exclusively on miRNAs with established mechanistic involvement in key signaling pathways relevant to pancreatic cancer biology, including KRAS, TP53, and CDKN2A. Each miRNA was linked to the pathway in which it is functionally implicated, followed by its observed specificity category derived from our pooled meta-analytic data. For instance, miR-34a, a known transcriptional target of TP53, demonstrated moderate specificity, while miR-155, which regulates both the NF-κB and PI3K/AKT pathways, was associated with high specificity. Although miR-145 and miR-125b are involved in tumor suppressive signaling, they were only reported in one or two studies and therefore remain underrepresented despite their mechanistic importance. Among the signaling pathways represented in **Figure 8B**, the PI3K/AKT/mTOR axis emerged as the most enriched, encompassing five miRNAs (i.e., miR-21, miR-155, miR-221, miR-223, and miR-20a). These miRNAs are functionally linked to cellular proliferation, apoptosis resistance, and survival signaling. The EMT/metastasis program was also well represented, including miR-10b, miR-21, miR-25, and miR-30c, all of which have been implicated in epithelial-mesenchymal transition (EMT) and cancer dissemination. Additional pathways included the NF-κB pathway (miR-21, miR-155, miR-223), the Wnt/β-catenin pathway (miR-34a, miR-92a, miR-194), and the KRAS pathway (miR-21, miR-193b). Pathways related to TP53 (miR-34a, miR-192) and CDKN2A/p16 (miR-221) were less populated, but still represented by biologically relevant miRNAs. Although SMAD4-TGFβ signaling is a critical tumor suppressor pathway in pancreatic cancer, none of the miRNAs known to regulate this axis (e.g., miR-130a, miR-421, miR-494) were reported across the included studies and were therefore not included in **Figure 8B**. This likely reflects a gap in the current biomarker literature rather than biological insignificance.

Taken together, these visual analyses highlight a subset of circulating miRNAs that not only show consistent diagnostic performance across studies but also align with known molecular mechanisms of pancreatic tumorigenesis. This layered approach offers a clearer rationale for prioritizing miRNAs in future translational studies, particularly those that converge on survival, inflammatory, and metastatic signaling pathways.

## 4 Discussion

Pancreatic cancer is one of the most lethal malignancies, largely due to its asymptomatic nature in the early stages, which makes timely diagnosis extremely challenging (3). By the time symptoms appear, the disease is often at an advanced stage, limiting treatment options and reducing survival rates. Therefore, the development of reliable, non-invasive diagnostic methods for early detection is of paramount importance (53). Liquid biopsy, utilizing blood or saliva as a source of biomarkers, represents a promising solution for early detection, offering a minimally invasive alternative to traditional tissue biopsies (26, 27, 48). In this context, circulating miRNAs have emerged as highly valuable biomarkers, capable of reflecting tumor presence and progression.

This meta-analysis represents the most comprehensive study to date, incorporating 168 sub-studies and a total of 1,496 patients, making it the largest comparative analysis of blood-derived miRNAs, saliva-derived miRNAs, and their combined use for pancreatic cancer detection. Notably, this study includes 119 sub-studies on saliva-derived miRNAs, the highest number reported in the field, making it the first meta-analysis to directly compare the diagnostic performance of blood-based, saliva-based, and combined miRNA biomarkers. By systematically evaluating their diagnostic potential, this study provides critical insights into their clinical utility and highlights the advantages of integrating both biofluids for pancreatic cancer detection.

Our findings confirm that blood-derived miRNAs exhibit strong diagnostic performance, with a pooled sensitivity of 0.83 (95% CI: 0.78-0.88) and specificity of 0.87 (95% CI: 0.82-0.91) (**Figure 3A**). The area under the curve (AUC) of 0.92 (95% CI: 0.89-0.94) reinforces their reliability as liquid biopsy biomarkers (**Figure 4A**). Furthermore, the diagnostic odds ratio (DOR) of 33.40 (95% CI: 17.88-62.37) indicates their effectiveness in distinguishing pancreatic cancer patients from non-cancer individuals (**Figure 6A**). These results validate the well-established role of blood-based miRNA testing as a highly accurate diagnostic tool. Similarly, saliva-derived miRNAs demonstrated excellent diagnostic capabilities, with a pooled sensitivity of 0.87 (95% CI: 0.84-0.90) and specificity of 0.86 (95% CI: 0.82-0.89) (**Figure 3B**). Notably, the AUC for saliva-derived miRNAs was slightly higher at 0.93 (95% CI: 0.90-0.95), suggesting that saliva-based tests may be at least as effective as blood-based assays, if not slightly superior (**Figure 4B**). Additionally, the DOR for saliva-derived miRNAs was 39.94 (95% CI: 28.66-55.67), surpassing that of blood-derived miRNAs, indicating a potential diagnostic advantage (**Figure 6B**). The lower diagnostic likelihood ratio negative (DLR negative) for saliva-derived miRNAs (0.15 vs. 0.19 for blood-derived miRNAs) further suggests that saliva-based testing may reduce false-negative rates, a crucial factor for early pancreatic cancer detection (**Figures 5A, B**). The combined analysis of blood- and saliva-derived miRNAs revealed a sensitivity of 0.86 (95% CI: 0.84-0.89) and specificity of 0.85 (95% CI: 0.83-0.88), with an AUC of 0.92 (95% CI: 0.90-0.94) (**Supplementary Figure 1**, **Figure 4C**). The DOR of 37.04 (95% CI: 27.66-49.60) suggests that integrating both biofluids may offer an additional diagnostic advantage by capturing complementary miRNA profiles (**Supplementary Figure 3**). This approach provides a broader representation of disease-associated miRNAs, as certain biomarkers may be differentially expressed in blood and saliva. Consequently, combining both sources could enhance diagnostic accuracy and robustness across diverse clinical scenarios.

miRNAs are small, non-coding RNA molecules that play a pivotal role in post-transcriptional gene regulation (54). Their biogenesis involves transcription into primary miRNAs (pri-miRNAs), processing into precursor miRNAs (pre-miRNAs), and further cleavage by the Dicer enzyme to generate mature miRNAs (55). Once incorporated into the RNA-induced silencing complex (RISC), they regulate gene expression through mRNA degradation or translational repression (55). Under normal physiological conditions, miRNAs govern critical cellular functions such as differentiation, proliferation, apoptosis, and immune responses (56, 57). However, in cancer, miRNA expression is frequently dysregulated, promoting tumor progression, metastasis, and therapy resistance (58). Some miRNAs function as tumor suppressors by inhibiting oncogene expression, while others act as oncomiRs, downregulating tumor suppressor genes (42). This dysregulation is a hallmark of cancer and supports the value of miRNAs as liquid biopsy biomarkers.

Given this dual biological role of miRNAs in both tumor suppression and oncogenesis, understanding their diagnostic potential requires not only statistical validation but also mechanistic context. In this study, we addressed this need by integrating pooled diagnostic performance with biological pathway relevance through the use of layered Sankey diagrams (**Figure 8**). These visualizations provide a systems-level perspective on how specific circulating miRNAs operate at the intersection of diagnostic utility and pancreatic cancer biology. In **Figure 8A**, we prioritized miRNAs that were both frequently reported and biologically plausible, revealing a cluster of candidates, such as miR-21, miR-196a, miR-92a, and miR-1246, with moderate-to-high diagnostic performance. miR-21 was not only the most frequently studied miRNA but also consistently associated with moderate sensitivity and specificity, reinforcing its candidacy as a robust diagnostic marker. Its established roles in KRAS and PI3K/AKT signaling further support its relevance in pancreatic tumorigenesis. Other miRNAs, such as miR-34a and miR-155, were included despite their lower frequency because of their strong functional ties to TP53 and inflammatory signaling, respectively, highlighting the importance of balancing study frequency with biological significance in biomarker prioritization.

**Figure 8B** extended this analysis by explicitly linking each miRNA to known signaling pathways implicated in pancreatic cancer. Among the pathways represented, PI3K/AKT/mTOR was the most enriched, comprising miR-21, miR-155, miR-221, miR-223, and miR-20a, all of which have been implicated in survival signaling and chemoresistance. Pathways related to EMT and metastasis, NF-κB, and Wnt/β-catenin were also represented, indicating that many of the top circulating miRNAs converge on processes related to invasion, inflammation, and cell survival. In contrast, tumor suppressor pathways such as TP53 and CDKN2A/p16 were represented by fewer miRNAs, and SMAD4-TGFβ signaling, despite its established relevance in pancreatic cancer, was not reflected in our figure due to the limited reporting of related miRNAs in circulating biomarker studies. This distribution highlights an important gap in the literature: while miRNAs associated with oncogenic and inflammatory signaling are frequently studied and consistently show diagnostic value, miRNAs involved in canonical tumor suppressor networks remain underrepresented in the circulating miRNA field. This may be attributable to technical detection biases or to the lower abundance of tumor-suppressive miRNAs in blood-derived compartments. Taken together, these findings emphasize the importance of integrating biological context into diagnostic meta-analyses. Visual frameworks like Sankey plots not only enhance interpretability of pooled results but also help identify promising, pathway-informed miRNA candidates for future validation. Our analysis suggests that miRNAs converging on PI3K/AKT, NF-κB, and EMT-related signaling hold particular promise for translational application, whereas mechanistically important yet infrequently studied miRNAs should be prioritized in future experimental designs.

In cancer, miRNAs are released into circulation through passive leakage from apoptotic or necrotic cells and active secretion via exosomes (40). Exosomal miRNAs are particularly relevant because they are actively secreted by cancer cells and encapsulated within lipid bilayers, protecting them from enzymatic degradation and ensuring stability as biomarkers (37). These exosomes can travel systemically and interact with salivary gland epithelial cells through endocytosis or membrane fusion, enabling the transfer of tumor-derived miRNAs into saliva (45). This biological mechanism supports the feasibility of saliva-based diagnostics and explains the strong correlation between salivary miRNAs and pancreatic cancer. Further understanding of these exosomal trafficking pathways may open up valuable diagnostic opportunities beyond early detection. Specifically, salivary miRNAs may be leveraged in the future for real-time monitoring of disease progression, predicting metastatic potential, or guiding treatment selection based on molecular signatures that reflect tumor dynamics in a non-invasive manner.

Previous studies have demonstrated that salivary biomarkers reliably reflect systemic disease states, reinforcing saliva’s potential as a viable diagnostic biofluid (48, 59). Our findings further substantiate that saliva-derived miRNAs not only mirror the diagnostic performance of blood-derived miRNAs but may offer additional advantages due to their ease of collection, non-invasiveness, and potential for reducing false-negative results. Importantly, the combined use of blood- and saliva-derived miRNAs provides a promising strategy to maximize diagnostic performance by leveraging complementary molecular information.

Despite these promising findings, certain limitations must be acknowledged. Our analysis detected publication bias in saliva-derived miRNA studies (p < 0.01), while no significant bias was found in blood-derived (p = 0.78) or combined analyses (p = 0.35) (**Figure 7**). One plausible explanation is that saliva-based miRNA diagnostics remain a relatively nascent area compared to blood-based approaches, with fewer studies overall and a higher proportion of exploratory or proof-of-concept research. In such early-stage fields, there may be a greater tendency for investigators and journals to preferentially report positive or promising findings, especially given the novelty and clinical appeal of non-invasive diagnostics. Studies with null or negative results may be less likely to be submitted or accepted for publication, contributing to an imbalance in the published literature. Moreover, technical factors specific to saliva, such as lower RNA yield, variable exosomal content, and higher susceptibility to degradation or contamination, may result in inconsistent results across studies. These challenges may increase the likelihood that only studies with particularly strong or favorable diagnostic signals are published, while technically inconclusive studies remain unpublished. This bias suggests a tendency toward selective reporting of positive results in saliva studies, necessitating future research with balanced reporting of both positive and negative findings. In addition, study heterogeneity remains a concern, although a random-effects model was applied to mitigate this issue. The lack of standardized miRNA panels across studies further limits comparability, emphasizing the need for consensus on key miRNA signatures. Larger, more standardized cohorts are essential to validate these findings and confirm the clinical utility of blood- and saliva-derived miRNAs.

Notably, our study also highlights a broader challenge in the field: the absence of universally reproducible circulating miRNA biomarkers across studies. Despite pooling data from 168 sub-studies involving over 1,400 patients, no single miRNA consistently demonstrated both high diagnostic performance and cross-study reproducibility. This inconsistency likely arises from technical factors (e.g., differences in sample type, RNA extraction, and quantification platforms), biological variability (e.g., tumor stage, stromal interaction, host immune response), and selective reporting biases. These limitations are especially pronounced in circulating miRNA research, where inter-study variation is high and standardization remains limited. To partially address this, we incorporated two layered Sankey plots (**Figures 8A, B**) that visualize the diagnostic performance and biological relevance of individual miRNAs. Although no “universal marker” emerged, several miRNAs such as miR-21, 155, 223, and 34a were identified as both more frequently reported and mechanistically linked to critical pathways in pancreatic cancer, including KRAS, NF-kB, and PI3K/AKT. These miRNAs may not function as standalone markers but could serve as reliable components of multi-marker panels tailored for pancreatic cancer detection. By integrating empirical diagnostic data with functional annotations, the Sankey plots offer a systems-level perspective that improves biomarker interpretability and prioritization.

Future research should therefore prioritize large-scale, prospective multi-center studies that apply standardized protocols for sample processing, RNA extraction, and miRNA quantification. Particular attention should be given to pathway-informed candidate miRNAs with consistent biological relevance. For saliva-based diagnostics, developing optimized multi-miRNA panels, along with rigorous validation in independent cohorts, will be essential. Furthermore, economic feasibility analyses comparing saliva- and blood-based testing modalities are warranted to assess cost-effectiveness in real-world clinical settings. Until such standardization and validation efforts are realized, reproducibility will remain a critical barrier to the clinical translation of circulating miRNA biomarkers.

## 5 Conclusion

In conclusion, this meta-analysis provides compelling evidence that both blood- and saliva-derived miRNAs serve as highly effective biomarkers for pancreatic cancer detection. Saliva-derived miRNAs demonstrate comparable, if not superior, diagnostic performance to blood-derived miRNAs, offering a non-invasive and potentially more accessible diagnostic alternative. The combined assessment of both biofluids presents a promising strategy to enhance diagnostic accuracy by integrating complementary miRNA profiles. These findings indicate the potential of miRNA-based liquid biopsies to revolutionize pancreatic cancer diagnostics, emphasizing the need for further validation and standardization to facilitate clinical implementation.

## Data Availability

The original contributions presented in the study are included in the article/**Supplementary Material**. Further inquiries can be directed to the corresponding author.
