# Supplementary material for: Diagnostic value of integrating salivary and blood miRNAs for pancreatic cancer detection
Source: Front Oncol. 2025 Oct 20;15:1642727. doi: 10.3389/fonc.2025.1642727 (PMC12580116; doi:10.3389/fonc.2025.1642727)
Supplement: Supplementary file 1 [file DataSheet1.pdf]

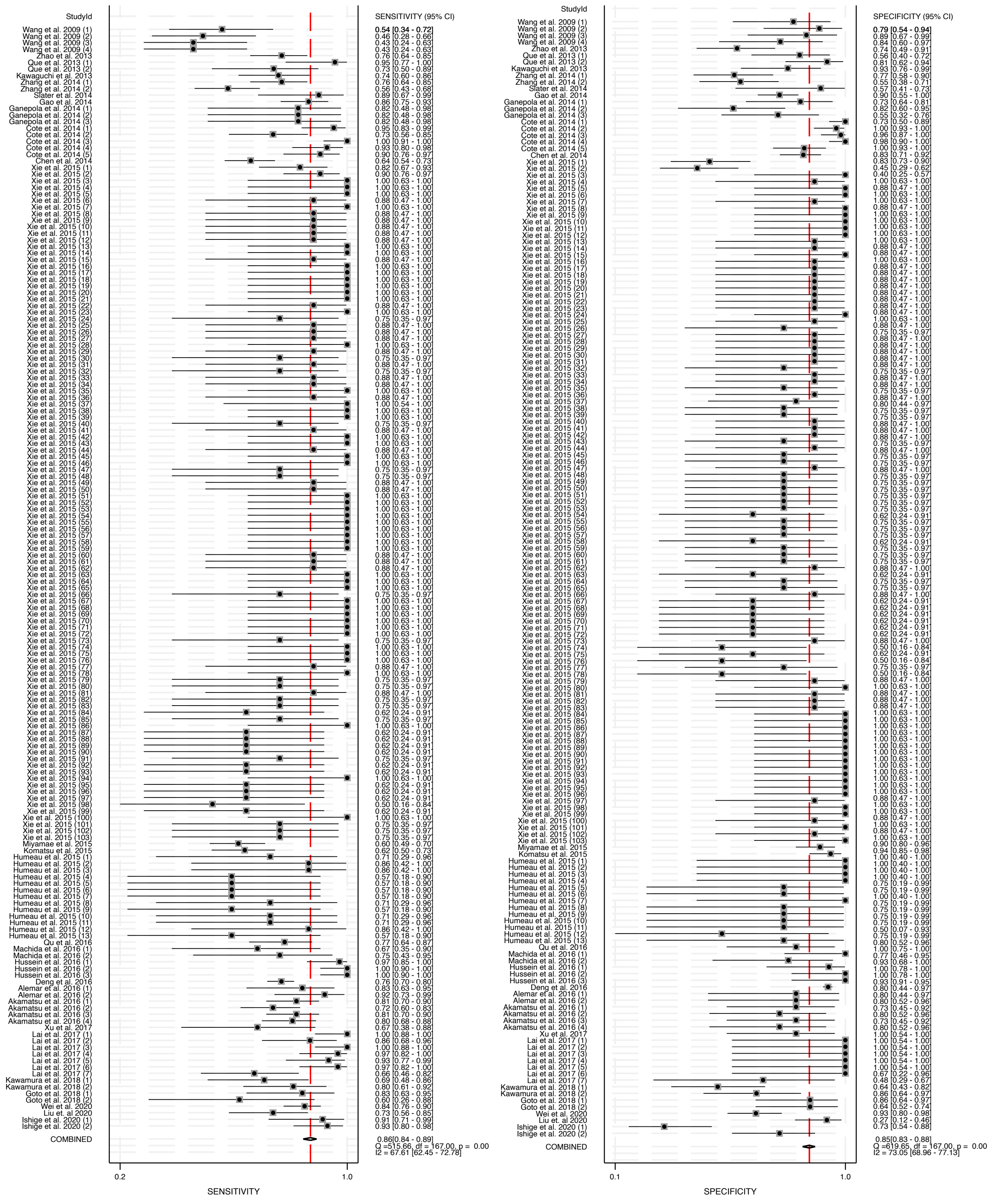

Supplementary Figure 1. Pooled sensitivity and specificity for combined blood- and saliva-derived miRNAs (Sensitivity: 0.86, Specificity: 0.85).

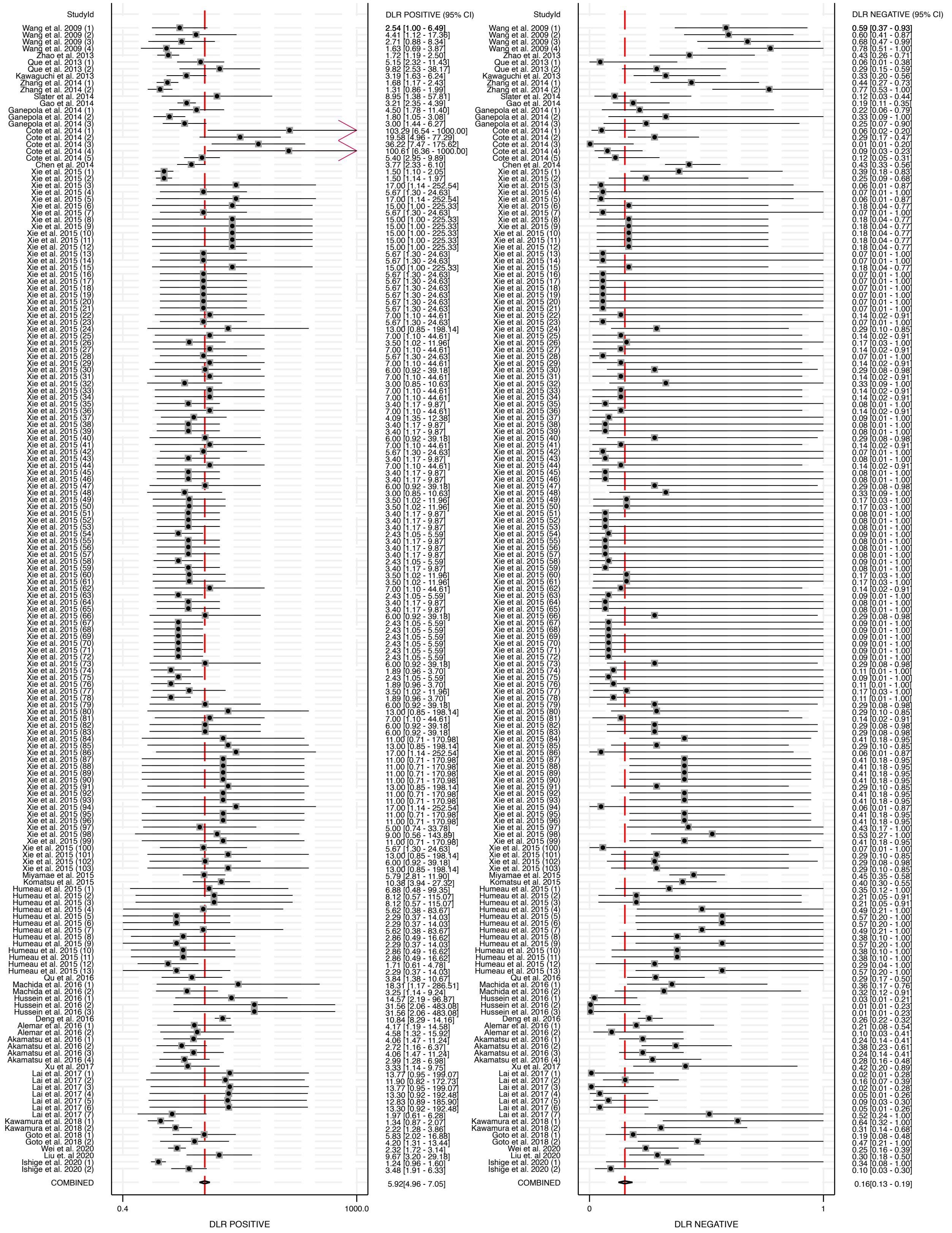

Supplementary Figure 2. Diagnostic likelihood ratios (DLRs) for combined blood- and saliva-derived miRNAs (DLR positive: 5.92, DLR negative: 0.16).

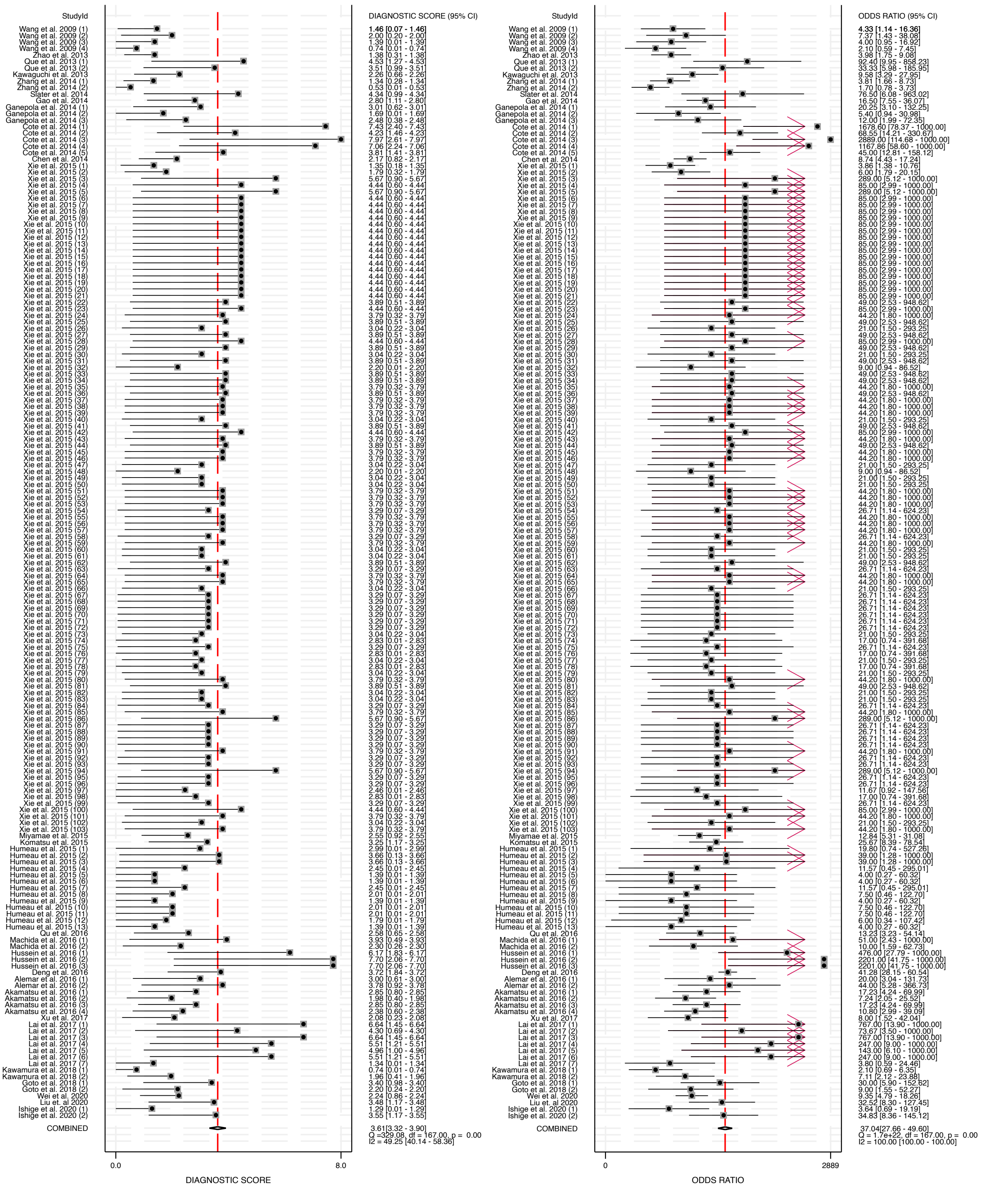

Supplementary Figure 3. Diagnostic score (DS) and diagnostic odds ratio (DOR) for combined blood- and saliva-derived miRNAs (DS: 3.61, DOR: 37.04).
