# Supplementary material for: Diagnostic value of integrating salivary and blood miRNAs for pancreatic cancer detection
Source: Front Oncol. 2025 Oct 20;15:1642727. doi: 10.3389/fonc.2025.1642727 (PMC12580116; doi:10.3389/fonc.2025.1642727)
Supplement: Supplementary file 2 [file DataSheet2.pdf]

SN	Study ID (Author, Year)	miRNA	Patient	Control	TP	FP	FN	TN	Sample	Ref
1	Ishige et al. 2020 (2)	miR-1246	41	30	38	8	3	22	Serum	(60)
2	Ishige et al. 2020 (1)	miR-1246	22	30	20	22	2	8	Saliva	(60)
3	Liu et. al 2020	miR-196a	40	40	29	3	11	37	Plasma	(61)
4	Wei et al. 2020	miR-1246	120	80	101	29	19	51	Serum	(62)
5	Goto et al. 2018 (2)	miR-21	10	21	6	3	4	18	Serum	(63)
6	Goto et al. 2018 (1)	miR-21	24	21	20	3	4	18	Serum	(63)
7	Kawamura et al. 2018 (2)	miR-21	30	25	24	9	6	16	Blood	(64)
8	Kawamura et al. 2018 (1)	miR-21	26	29	18	15	8	14	Blood	(64)
9	Lai et al. 2017 (7)	miR-483	29	6	19	2	10	4	Plasma	(65)
10	Lai et al. 2017(6)	miR-181a	29	6	28	0	1	6	Plasma	(65)
11	Lai et al. 2017 (5)	miR-20a	29	6	27	0	2	6	Plasma	(65)
12	Lai et al. 2017(4)	miR-106b	29	6	28	0	1	6	Plasma	(65)
13	Lai et al. 2017 (3)	miR-30c	29	6	29	0	0	6	Plasma	(65)
14	Lai et al. 2017 (2)	miR-21	29	6	25	0	4	6	Plasma	(65)
15	Lai et al. 2017 (1)	miR-10b	29	6	29	0	0	6	Plasma	(65)
16	Xu et al. 2017	miR-1246	15	15	10	3	5	12	Plasma	(66)
17	Akamatsu et al. 2016 (4)	miR-193b	69	15	55	4	14	11	Serum	(67)
18	Akamatsu et al. 2016 (3)	miR-181d	69	15	56	3	13	12	Serum	(67)
19	Akamatsu et al. 2016 (2)	miR-7	69	15	50	4	19	11	Serum	(67)
20	Akamatsu et al. 2016 (1)	miR-34a	69	15	56	3	13	12	Serum	(67)
21	Alemar et al. 2016 (2)	miR-34a	24	10	22	2	2	8	Serum	(68)
22	Alemar et al. 2016 (1)	miR-21	24	10	20	2	4	8	Serum	(68)
23	Deng et al. 2016	miR-25	303	760	229	53	74	707	Serum	(69)
24	Hussein et al. 2016 (3)	miR-885	35	15	35	0	0	15	Plasma	(70)
25	Hussein et al. 2016(2)	miR-642b	35	15	35	0	0	15	Plasma	(70)
26	Hussein et al. 2016 (1)	miR-22	35	15	34	1	1	14	Plasma	(70)
27	Machida et al. 2016 (2)	miR-4644	12	13	9	3	3	10	Saliva	(71)
28	Machida et al. 2016 (1)	miR-1246	12	13	8	0	4	13	Saliva	(71)
29	Qu et al. 2016	miR-21	56	15	43	3	13	12	Serum	(72)
30	Humeau et al. 2015 (13)	miR-223	7	4	4	1	3	3	Saliva	(73)
31	Humeau et al. 2015 (12)	miR-222	7	4	6	2	1	2	Saliva	(73)
32	Humeau et al. 2015 (11)	miR-127-5p	7	4	5	1	2	3	Saliva	(73)
33	Humeau et al. 2015 (10)	miR-205	7	4	5	1	2	3	Saliva	(73)
34	Humeau et al. 2015 (9)	miR-20a	7	4	4	1	3	3	Saliva	(73)
35	Humeau et al. 2015 (8)	miR-92a	7	4	5	1	2	3	Saliva	(73)
36	Humeau et al. 2015 (7)	miR-92a	7	4	4	0	3	4	Saliva	(73)
37	Humeau et al. 2015 (6)	miR-25	7	4	4	1	3	3	Saliva	(73)
38	Humeau et al. 2015 (5)	miR-203	7	4	4	1	3	3	Saliva	(73)
39	Humeau et al. 2015 (4)	miR-29c	7	4	4	0	3	4	Saliva	(73)
40	Humeau et al. 2015 (3)	miR-23b	7	4	6	0	1	4	Saliva	(73)
41	Humeau et al. 2015 (2)	miR-23a	7	4	6	0	1	4	Saliva	(73)
42	Humeau et al. 2015 (1)	miR-21	7	4	5	0	2	4	Saliva	(73)
43	Komatsu et al. 2015	miR-223	94	68	44	4	27	63	Plasma	(74)
44	Miyamae et al. 2015	miR-744	94	68	56	7	38	61	Plasma	(75)
45	Xie et al. 2015 (103)	miR-3162-3p	8	8	6	0	2	8	Saliva	(76)
46	Xie et al. 2015 (102)	miR-1229-5p	8	8	6	1	2	7	Saliva	(76)
47	Xie et al. 2015 (101)	miR-6068	8	8	6	0	2	8	Saliva	(76)
48	Xie et al. 2015 (100)	miR-4507	8	8	8	1	0	7	Saliva	(76)
49	Xie et al. 2015 (99)	miR-6124	8	8	5	0	3	8	Saliva	(76)
50	Xie et al. 2015 (98)	miR-1202	8	8	4	0	4	8	Saliva	(76)
51	Xie et al. 2015 (97)	miR-4466	8	8	5	1	3	7	Saliva	(76)
52	Xie et al. 2015 (96)	miR-4454	8	8	5	0	3	8	Saliva	(76)

53	Xie et al. 2015 (95)	miR-4763-3p	8	8	5	0	3	8	Saliva	(76)
54	Xie et al. 2015 (94)	miR-3676-3p	8	8	8	0	0	8	Saliva	(76)
55	Xie et al. 2015 (93)	miR-2861	8	8	5	0	3	8	Saliva	(76)
56	Xie et al. 2015 (92)	miR-197-5p	8	8	5	0	3	8	Saliva	(76)
57	Xie et al. 2015 (91)	miR-1290	8	8	6	0	2	8	Saliva	(76)
58	Xie et al. 2015 (90)	miR-4687-3p	8	8	5	0	3	8	Saliva	(76)
59	Xie et al. 2015 (89)	miR-3665	8	8	5	0	3	8	Saliva	(76)
60	Xie et al. 2015 (88)	miR-6515-3p	8	8	5	0	3	8	Saliva	(76)
61	Xie et al. 2015 (87)	miR-1915-3p	8	8	5	0	3	8	Saliva	(76)
62	Xie et al. 2015 (86)	miR-4649-3p	8	8	8	0	0	8	Saliva	(76)
63	Xie et al. 2015 (85)	miR-191-3p	8	8	6	0	2	8	Saliva	(76)
64	Xie et al. 2015 (84)	miR-638	8	8	5	0	3	8	Saliva	(76)
65	Xie et al. 2015 (83)	miR-6076	8	8	6	1	2	7	Saliva	(76)
66	Xie et al. 2015 (82)	miR-1246	8	8	6	1	2	7	Saliva	(76)
67	Xie et al. 2015 (81)	miR-4788	8	8	7	1	1	7	Saliva	(76)
68	Xie et al. 2015 (80)	miR-4530	8	8	6	0	2	8	Saliva	(76)
69	Xie et al. 2015 (79)	miR-1228-3p	8	8	6	1	2	7	Saliva	(76)
70	Xie et al. 2015 (78)	miR-203a	8	8	8	4	0	4	Saliva	(76)
71	Xie et al. 2015 (77)	miR-939-5p	8	8	7	2	1	6	Saliva	(76)
72	Xie et al. 2015 (76)	miR-24-3p	8	8	8	4	0	4	Saliva	(76)
73	Xie et al. 2015 (75)	miR-92a-3p	8	8	8	3	0	5	Saliva	(76)
74	Xie et al. 2015 (74)	miR-15a-5p	8	8	8	4	0	4	Saliva	(76)
75	Xie et al. 2015 (73)	miR-4271	8	8	6	1	2	7	Saliva	(76)
76	Xie et al. 2015 (72)	miR-211-3p	8	8	8	3	0	5	Saliva	(76)
77	Xie et al. 2015 (71)	miR-3648	8	8	8	3	0	5	Saliva	(76)
78	Xie et al. 2015 (70)	miR-557	8	8	8	3	0	5	Saliva	(76)
79	Xie et al. 2015 (69)	miR-663a	8	8	8	3	0	5	Saliva	(76)
80	Xie et al. 2015 (68)	miR-575	8	8	8	3	0	5	Saliva	(76)
81	Xie et al. 2015 (67)	miR-134	8	8	8	3	0	5	Saliva	(76)
82	Xie et al. 2015 (66)	miR-1268b	8	8	6	1	2	7	Saliva	(76)
83	Xie et al. 2015 (65)	miR-1185-1-3p	8	8	8	2	0	6	Saliva	(76)
84	Xie et al. 2015 (64)	miR-6723-5p	8	8	8	2	0	6	Saliva	(76)
85	Xie et al. 2015 (63)	miR-1260a	8	8	8	3	0	5	Saliva	(76)
86	Xie et al. 2015 (62)	miR-6126	8	8	7	1	1	7	Saliva	(76)
87	Xie et al. 2015 (61)	miR-6722-3p	8	8	7	2	1	6	Saliva	(76)
88	Xie et al. 2015 (60)	miR-1275	8	8	7	2	1	6	Saliva	(76)
89	Xie et al. 2015 (59)	miR-345-3p	8	8	8	2	0	6	Saliva	(76)
90	Xie et al. 2015 (58)	miR-5006-5p	8	8	8	3	0	5	Saliva	(76)
91	Xie et al. 2015 (57)	miR-320a	8	8	8	2	0	6	Saliva	(76)
92	Xie et al. 2015 (56)	miR-4738-3p	8	8	8	2	0	6	Saliva	(76)
93	Xie et al. 2015 (55)	miR-4655-5p	8	8	8	2	0	6	Saliva	(76)
94	Xie et al. 2015 (54)	miR-4721	8	8	8	3	0	5	Saliva	(76)
95	Xie et al. 2015 (53)	miR-4707-5p	8	8	8	2	0	6	Saliva	(76)
96	Xie et al. 2015 (52)	miR-4778-5p	8	8	8	2	0	6	Saliva	(76)
97	Xie et al. 2015 (51)	miR-4419a	8	8	8	2	0	6	Saliva	(76)
98	Xie et al. 2015 (50)	miR-320c	8	8	7	2	1	6	Saliva	(76)
99	Xie et al. 2015 (49)	miR-3141	8	8	7	2	1	6	Saliva	(76)
100	Xie et al. 2015 (48)	miR-4664-3p	8	8	6	2	2	6	Saliva	(76)
101	Xie et al. 2015 (47)	miR-6085	8	8	6	1	2	7	Saliva	(76)
102	Xie et al. 2015 (46)	miR-6086	8	8	8	2	0	6	Saliva	(76)
103	Xie et al. 2015 (45)	miR-1224-5p	8	8	8	2	0	6	Saliva	(76)
104	Xie et al. 2015 (44)	miR-3196	8	8	7	1	1	7	Saliva	(76)
105	Xie et al. 2015 (43)	miR-371a-5p	8	8	8	2	0	6	Saliva	(76)
106	Xie et al. 2015 (42)	miR-1273f	8	8	8	1	0	7	Saliva	(76)

107	Xie et al. 2015 (41)	miR-4433-5p	8	8	7	1	1	7	Saliva	(76)
108	Xie et al. 2015 (40)	miR-4787-5p	8	8	6	1	2	7	Saliva	(76)
109	Xie et al. 2015 (39)	miR-3937	8	8	8	2	0	6	Saliva	(76)
110	Xie et al. 2015 (38)	miR-765	8	8	8	2	0	6	Saliva	(76)
111	Xie et al. 2015 (37)	miR-4669	8	8	6	2	0	8	Saliva	(76)
112	Xie et al. 2015 (36)	miR-4534	8	8	7	1	1	7	Saliva	(76)
113	Xie et al. 2015 (35)	miR-4800-5p	8	8	8	2	0	6	Saliva	(76)
114	Xie et al. 2015 (34)	miR-4532	8	8	7	1	1	7	Saliva	(76)
115	Xie et al. 2015 (33)	miR-940	8	8	7	1	1	7	Saliva	(76)
116	Xie et al. 2015 (32)	miR-4499	8	8	6	2	2	6	Saliva	(76)
117	Xie et al. 2015 (31)	miR-4634	8	8	7	1	1	7	Saliva	(76)
118	Xie et al. 2015 (30)	miR-371b-5p	8	8	6	1	2	7	Saliva	(76)
119	Xie et al. 2015 (29)	miR-150-3p	8	8	7	1	1	7	Saliva	(76)
120	Xie et al. 2015 (28)	miR-4484	8	8	8	1	0	7	Saliva	(76)
121	Xie et al. 2015 (27)	miR-3188	8	8	7	1	1	7	Saliva	(76)
122	Xie et al. 2015 (26)	miR-630	8	8	7	2	1	6	Saliva	(76)
123	Xie et al. 2015 (25)	miR-2392	8	8	7	1	1	7	Saliva	(76)
124	Xie et al. 2015 (24)	miR-5001-5p	8	8	6	0	2	8	Saliva	(76)
125	Xie et al. 2015 (23)	miR-572	8	8	8	1	0	7	Saliva	(76)
126	Xie et al. 2015 (22)	miR-937-5p	8	8	7	1	1	7	Saliva	(76)
127	Xie et al. 2015 (21)	miR-5703	8	8	8	1	0	7	Saliva	(76)
128	Xie et al. 2015 (20)	miR-483-5p	8	8	8	1	0	7	Saliva	(76)
129	Xie et al. 2015 (19)	miR-4665-3p	8	8	8	1	0	7	Saliva	(76)
130	Xie et al. 2015 (18)	miR-3135b	8	8	8	1	0	7	Saliva	(76)
131	Xie et al. 2015 (17)	miR-1227-5p	8	8	8	1	0	7	Saliva	(76)
132	Xie et al. 2015 (16)	miR-3156-5p	8	8	8	1	0	7	Saliva	(76)
133	Xie et al. 2015 (15)	miR-4433-3p	8	8	7	0	1	8	Saliva	(76)
134	Xie et al. 2015 (14)	miR-1914-3p	8	8	8	1	0	7	Saliva	(76)
135	Xie et al. 2015 (13)	miR-4632-5p	8	8	8	1	0	7	Saliva	(76)
136	Xie et al. 2015 (12)	miR-5100	8	8	7	0	1	8	Saliva	(76)
137	Xie et al. 2015 (11)	miR-3940-5p	8	8	7	0	1	8	Saliva	(76)
138	Xie et al. 2015 (10)	miR-4257	8	8	7	0	1	8	Saliva	(76)
139	Xie et al. 2015 (9)	miR-3676-5p	8	8	7	0	1	8	Saliva	(76)
140	Xie et al. 2015 (8)	miR-3679-5p	8	8	7	0	1	8	Saliva	(76)
141	Xie et al. 2015 (7)	miR-1273g-3p	8	8	8	1	0	7	Saliva	(76)
142	Xie et al. 2015 (6)	miR-4327	8	8	7	0	1	8	Saliva	(76)
143	Xie et al. 2015 (5)	miR-22-3p	8	8	8	0	0	8	Saliva	(76)
144	Xie et al. 2015 (4)	miR-4442	8	8	8	1	0	7	Saliva	(76)
145	Xie et al. 2015 (3)	miR-1587	8	8	8	0	0	8	Saliva	(76)
146	Xie et al. 2015 (2)	miR-940	40	40	36	24	4	16	Saliva	(76)
147	Xie et al. 2015 (1)	miR-3679-5p	40	40	33	22	7	18	Saliva	(76)
148	Chen et al. 2014	miR-182	109	88	70	15	39	73	Plasma	(77)
149	Cote et al. 2014 (5)	miR-212	40	54	36	9	4	45	Plasma	(78)
150	Cote et al. 2014 (4)	miR-155	40	54	37	0	3	54	Plasma	(78)
151	Cote et al. 2014 (3)	miR-106b	40	54	40	1	0	53	Plasma	(78)
152	Cote et al. 2014 (2)	miR-30c	40	54	29	2	11	52	Plasma	(78)
153	Cote et al. 2014 (1)	miR-10b	40	54	38	0	2	54	Plasma	(78)
154	Ganepola et al. 2014 (3)	miR-885	11	22	9	6	2	16	Plasma	(79)
155	Ganepola et al. 2014 (2)	miR-642	11	22	9	10	2	12	Plasma	(79)
156	Ganepola et al. 2014 (1)	miR-22	11	22	9	4	2	18	Plasma	(79)
157	Gao et al. 2014	miR-16	70	120	60	32	10	88	Plasma	(80)
158	Slater et al. 2014	miR-196a	19	10	17	1	2	9	Serum	(81)
159	Zhang et al. 2014 (2)	miR-194	70	40	39	17	31	23	Serum	(82)
160	Zhang et al. 2014 (1)	miR-192	70	40	53	18	17	22	Serum	(82)

161	Kawaguchi et al. 2013	miR-221	47	30	35	7	12	23	Plasma	(83)
162	Que et al. 2013 (2)	miR-17	22	27	16	2	6	25	Serum	(84)
163	Que et al. 2013 (1)	miR-21	22	27	21	5	1	22	Serum	(84)
164	Zhao et al. 2013	miR-192	70	40	53	18	17	23	Serum	(85)
165	Wang et al. 2009 (4)	miR-210	28	19	12	5	16	14	Plasma	(86)
166	Wang et al. 2009 (3)	miR-196a	28	19	12	3	16	16	Plasma	(86)
167	Wang et al. 2009 (2)	miR-21	28	19	13	2	15	17	Plasma	(86)
168	Wang et al. 2009 (1)	miR-155	28	19	15	4	13	15	Plasma	(86)

Supplementary Table 1. Characteristics of the 168 sub-studies included in the meta-analysis. Numerical designations were appended to the publication year to distinguish multiple sub-studies from the same paper. The miRNA panels analyzed in Xie et al. 2015 consist of 103 miRNAs, the details of which are presented in this table. SN, Serial number.

Author, Year	Location	Cohort	miRNA type	Age (mean)	M:F ratio	Cancer type	Stage	Ref
Ishige et al. 2020	Japan	Hospital	Circulating	68.1	25:16	PDAC, IPMN	0-IV	(60)
Liu et. al 2020	China	Hospital	Circulating	58.0	22:!8	PDAC	I-IV	(61)
Wei et al. 2020	China	Hospital	Circulating	62.7	70:50	PC	I-IV	(62)
Goto et al. 2018	Japan	Hospital	Circulating	64.0	17:15	PC	I-IV	(63)
Kawamura et al. 2018	Japan	Hospital	Exosomal	67.0	33:22	PDAC	I-II	(64)
Lai et al. 2017	USA	Hospital	Exosomal	67.3	15:14	PDAC	I-IV	(65)
Xu et al. 2017	USA	Hospital	Exosomal	66.6	8:7	PDAC, IPMN, NET	I-II	(66)
Akamatsu et al. 2016	Japan	Hospital	Circulating	68.2	45:24	PDAC	I-IV	(67)
Alemar et al. 2016	Brazil	Hospital	Circulating	62.0	12:12	PDAC	I-IV	(68)
Deng et al. 2016	China	Hospital	Circulating	62.0	188:115	PC	I-IV	(69)
Hussein et al. 2016	Egypt	Hospital	Circulating	57.4	14:21	PDAC	I-IV	(70)
Machida et al. 2016	Japan	Hospital	Exosomal	65.0	6:6	PC	II-IV	(71)
Qu et al. 2016	China	Hospital	Circulating	52.0	58:37	PC	I-IV	(72)
Humeau et al. 2015	France	Hospital	Circulating	67.0	N/A	PDAC	N/A	(73)
Komatsu et al. 2015	Japan	Hospital	Circulating	N/A	41:30	PIDC, IPMN	II-IV	(74)
Miyamae et al. 2015	Japan	Hospital	Circulating	51.9	52:42	IPMN, PDAC	I-IV	(75)
Xie et al. 2015	China	Hospital	Circulating	63.5	3:5	PDAC, MCN	I-III	(76)
Chen et al. 2014	China	Hospital	Circulating	N/A	40:69	PC	I-IV	(77)
Cote et al. 2014	USA	Hospital	Circulating	66.5	16:24	PDAC	I-IV	(78)
Ganepola et al. 2014	USA	Hospital	Circulating	68.0	6:5	PDAC	II	(79)
Gao et al. 2014	China	Hospital	Circulating	49.0	123/67	PC	N/A	(80)
Slater et al. 2014	Germany	Hospital	Circulating	N/A	N/A	PC	I-IV	(81)
Zhang et al. 2014	China	Hospital	Circulating	61.0	15:16	PDAC	N/A	(82)
Kawaguchi et al. 2013	Japan	Hospital	Circulating	N/A	27:20	PDAC	0-IV	(83)
Que et al. 2013	China	Hospital	Exosomal	65.3	16:6	PDAC	I-IV	(84)
Zhao et al. 2013	China	Hospital	Circulating	N/A	42:28	PDAC	I-IV	(85)
Wang et al. 2009	USA	Hospital	Circulating	N/A	25:24	PDAC	I-IV	(86)

Supplementary Table 2. Patient demographics of the studies included in the meta-analysis. PDAC, pancreatic ductal adenocarcinoma; IPMN, intraductal papillary mucinous neoplasm; PC, pancreatic cancer; NET, neuroendocrine tumors; PIDC, pancreatic invasive ductal carcinoma; MCN, mucinous cystic neoplasm; N/A, not applicable.
